# Expression of SRP-9001 dystrophin and stabilization of motor function up to 2 years post-treatment with delandistrogene moxeparvovec gene therapy in individuals with Duchenne muscular dystrophy

**DOI:** 10.3389/fcell.2023.1167762

**Published:** 2023-07-11

**Authors:** Jerry R. Mendell, Perry B. Shieh, Craig M. McDonald, Zarife Sahenk, Kelly J. Lehman, Linda P. Lowes, Natalie F. Reash, Megan A. Iammarino, Lindsay N. Alfano, Brenna Sabo, Jeremy D. Woods, Christy L. Skura, Howard C. Mao, Loretta A. Staudt, Danielle A. Griffin, Sarah Lewis, Shufang Wang, Rachael A. Potter, Teji Singh, Louise R. Rodino-Klapac

**Affiliations:** ^1^ Center for Gene Therapy, Abigail Wexner Research Institute, Nationwide Children’s Hospital, Columbus, OH, United States; ^2^ Department of Pediatrics, The Ohio State University, Columbus, OH, United States; ^3^ Department of Neurology, The Ohio State University, Columbus, OH, United States; ^4^ UCLA Medical Center, Los Angeles, CA, United States; ^5^ Departments of Physical Medicine and Rehabilitation and Pediatrics, Lawrence J. Ellison Ambulatory Care Center, UC Davis Health, Sacramento, CA, United States; ^6^ Sarepta Therapeutics Inc, Cambridge, MA, United States

**Keywords:** Duchenne muscular dystrophy, gene therapy, dystrophin, AAVrh74, SRP-9001

## Abstract

**Introduction:** Delandistrogene moxeparvovec (SRP-9001) is an investigational gene transfer therapy designed for targeted expression of SRP-9001 dystrophin protein, a shortened dystrophin retaining key functional domains of the wild-type protein.

**Methods:** This Phase 2, double-blind, two-part (48 weeks per part) crossover study (SRP-9001-102 [Study 102]; NCT03769116) evaluated delandistrogene moxeparvovec in patients, aged ≥4 to <8 years with Duchenne muscular dystrophy. Primary endpoints (Part 1) were change from baseline (CFBL) in SRP-9001 dystrophin expression (Week 12), by Western blot, and in North Star Ambulatory Assessment (NSAA) score (Week 48). Safety assessments included treatment-related adverse events (TRAEs). Patients were randomized and stratified by age to placebo (n = 21) or delandistrogene moxeparvovec (n = 20) and crossed over for Part 2.

**Results:** SRP-9001 dystrophin expression was achieved in all patients: mean CFBL to Week 12 was 23.82% and 39.64% normal in Parts 1 and 2, respectively. In Part 1, CFBL to Week 48 in NSAA score (least-squares mean, LSM [standard error]) was +1.7 (0.6) with treatment versus +0.9 (0.6) for placebo; *p* = 0.37. Disparity in baseline motor function between groups likely confounded these results. In 4- to 5-year-olds with matched baseline motor function, CFBL to Week 48 in NSAA scores was significantly different (+2.5 points; *p* = 0.0172), but not significantly different in 6-to-7-year-olds with imbalanced baseline motor function (−0.7 points; *p* = 0.5384). For patients treated with delandistrogene moxeparvovec in Part 2, CFBL to Week 48 in NSAA score was +1.3 (2.7), whereas for those treated in Part 1, NSAA scores were maintained. As all patients in Part 2 were exposed to treatment, results were compared with a propensity-score-weighted external control (EC) cohort. The LSM difference in NSAA score between the Part 2 treated group and EC cohort was statistically significant (+2.0 points; *p* = 0.0009). The most common TRAEs were vomiting, decreased appetite, and nausea. Most occurred within the first 90 days and all resolved.

**Discussion:** Results indicate robust expression of SRP-9001 dystrophin and overall stabilization in NSAA up to 2 years post-treatment. Differences in NSAA between groups in Part 1 were not significant for the overall population, likely because cohorts were stratified only by age, and other critical prognostic factors were not well matched at baseline.

## Introduction

Duchenne muscular dystrophy (DMD) is a rare, progressive, X-linked neuromuscular disease caused by the absence of functional dystrophin protein in skeletal, cardiac, and respiratory muscle due to mutations in the *DMD* gene (Ryder et al., 2017; Cowen et al., 2019). The lack of functional dystrophin protein leads to progressive muscle weakness, loss of ambulation, respiratory weakness, and cardiomyopathy (Cowen et al., 2019; Han et al., 2020). While the clinical symptoms of DMD typically manifest between 3 and 5 years of age (Cowen et al., 2019), characteristics of disease pathology, such as muscle damage, can be seen *in utero* (Guo, 1990).

Several therapies that can slow disease progression or extend survival are available for patients with DMD (Gloss et al., 2016; Mackenzie et al., 2021). Corticosteroid treatment, the standard of care in DMD, aims to treat the symptoms of DMD and slow disease progression; however, long-term use of corticosteroids is associated with significant side effects, including excessive weight gain, delayed growth, and osteoporosis (Gloss et al., 2016; Kourakis et al., 2021). In addition, this treatment is palliative only and does not address the absence of functional dystrophin protein, the underlying cause of this disease (Kourakis et al., 2021). Although there are several approved exon-skipping therapies, fewer than 30% of all patients with DMD have mutations that are amenable to these treatments. Furthermore, these modalities, while disease modifying, require chronic, lifelong administration (Birnkrant et al., 2018; Muntoni et al., 2019; Heo, 2020; Sun et al., 2020; Wilton-Clark and Yokota, 2023). Thus, there is an unmet need for disease-modifying treatments with broader patient applicability.

A promising approach for the treatment of DMD aims to restore production of functional dystrophin protein through adeno-associated virus (AAV)-mediated gene transfer therapy (Elangkovan and Dickson, 2021). Delandistrogene moxeparvovec is a recombinant AAV rhesus isolate serotype 74 (rAAVrh74)-based gene therapy in development for patients with DMD (Mendell et al., 2020). The aim of this treatment is to address the underlying cause of DMD through targeted expression of SRP-9001 dystrophin, a shortened dystrophin protein that retains the key functional domains of the wild-type protein, in skeletal and cardiac muscle (Mendell et al., 2020). Preclinical animal studies have demonstrated safety and efficacy following systemic delivery of delandistrogene moxeparvovec, supporting the initiation of Phase 1 clinical trials (Potter et al., 2021). SRP-9001-101 (Study 101; NCT03375164), a Phase 1/2a trial of delandistrogene moxeparvovec in four patients with DMD, demonstrated a favorable safety profile and robust protein expression. Sustained improvement and subsequent stabilization of motor function, measured using the North Star Ambulatory Assessment (NSAA) and timed function tests, were observed at 4 years post-treatment in patients with a mean age of 9.2 years, when a steep decline in motor function would be predicted based on natural history (Mendell et al. Manuscript in review).

Here, we report the results from SRP-9001-102 (Study 102; NCT03769116): a two-part, Phase 2, double-blind, placebo-controlled study evaluating the safety and efficacy of a single intravenous (IV) administration of delandistrogene moxeparvovec in patients with DMD aged ≥4 to <8 years (https://clinicaltrials.gov/ct2/show/NCT03769116). Due to the absence of a control comparator in Part 2 of this study, data from a propensity-score-weighted external control (EC) cohort were used to contextualize the Part 2 functional results.

## Materials and methods

### Ethics statement

This study was approved by an internal review board at Sarepta Therapeutics, Inc., Cambridge, MA, United States in line with the Declaration of Helsinki and principles of Good Clinical Practice. The trial was approved by the institutional review boards of participating sites. Signed informed consent was obtained from participants’ parents, in compliance with the Code of Federal Regulations, Title 21, Part 50, and International Conference on Harmonization guidelines.

### Study design and participants

This study is a Phase 2, randomized, double-blind, placebo-controlled crossover study with an ongoing open-label extension. Following a screening period of up to 4 weeks, participants were randomized 1:1 by Interactive Voice/Web Response System to receive a single IV administration of either delandistrogene moxeparvovec or placebo (up to 10 mL/kg of lactated Ringer’s solution). Upon completion of the first 48-week period (Part 1), participants were crossed over to the corresponding treatment group to receive either placebo or delandistrogene moxeparvovec during the second 48-week period (Part 2). To maintain blinding, participants who were randomized to receive delandistrogene moxeparvovec in Part 1 received up to 10 mL/kg of placebo in Part 2. Randomization was stratified by age at baseline (4–5 vs 6–7 years), while functional measures were not included. The final study visit will be at Week 212 or 260 for patients who received delandistrogene moxeparvovec in Part 1 or 2, respectively.

One day prior to infusion (placebo or delandistrogene moxeparvovec), the background dose of steroid was increased to ≥1 mg/kg of a glucocorticoid (prednisone equivalent) daily; this increased dose was continued for ≥60 days post-infusion, unless earlier tapering was judged by the study investigator to be in the patient’s best interest. Muscle biopsies (gastrocnemius or other muscle selected by the study investigator) were performed at screening/baseline, Week 12 of Part 1, and Week 12 of Part 2 (or no later than Week 48). Muscle biopsies were used to quantify the following: SRP-9001 vector genome (vg) copies using polymerase chain reaction (PCR); SRP-9001 dystrophin expression by Western blot; and SRP-9001 dystrophin localization and quantification by immunofluorescence (IF) fiber intensity and IF percent dystrophin-positive fibers (PDPF).

The study was initiated in December 2018. Forty-one patients were screened and enrolled at two sites in the United States: Nationwide Children’s Hospital in Columbus, Ohio, and the University of California, Los Angeles, California. The target sample size was calculated based on 90% power to detect a difference between the treatment and placebo groups in the least-squares mean (LSM) change from baseline (CFBL) to Week 48 in NSAA score of 5 points, with a standard deviation of 5, and a 2-sided, Type 1 error of 0.05. Eligibility criteria included: ≥4 to <8 years of age at the time of screening in Part 1; a confirmed mutation in the *DMD* gene (frameshift [deletion or duplication] or premature stop codon mutation) between exons 18 and 58 to limit potential immune responses to the transgene (Mendell et al., 2010); creatine kinase >1,000 U/L; percent predicted 100-m Walk/Run (100MWR) time <95th; ability to cooperate with the motor assessment testing; and on a stable dose of oral corticosteroids for ≥12 weeks prior to screening. Key exclusion criteria included: treatment with an investigational medicine ≤6 months prior to screening; impaired cardiovascular function on echocardiogram (ECHO); presence of any significant genetic disease other than DMD; physical examination, ECHO, or laboratory findings that could adversely affect participant safety, compromise completion of follow-up, or impair assessment of study results; severe infection ≤4 weeks before treatment; and rAAVrh74 antibody titers >1:400, as determined by the Genetic Therapies Center of Excellence enzyme-linked immunosorbent assay. A full list of inclusion and exclusion criteria can be found in the Supplementary Material.

### Vector production and dosing procedures

Vector production and dosing procedures have been previously described (Mendell et al., 2020). Briefly, a quantitative PCR (qPCR)-based titration method based on a supercoiled plasmid standard was used to determine an encapsulated vg titer utilizing a Prism 7,500 Fast TaqMan detector system (PE Applied Biosystems). The qPCR titer method uses a vector-specific primer probe set for sequences of the MHCK7 promoter (within the delandistrogene moxeparvovec gene cassette) (Schnepp et al., 2005). All patients in Part 1 treated with delandistrogene moxeparvovec received 2.0 × 10^14^ vg/kg of clinical process material, as determined by the supercoiled standard qPCR. The 2.0 × 10^14^ vg/kg dose, estimated by supercoiled standard qPCR, was subsequently found to be equivalent to a 1.33 × 10^14^ vg/kg dose by linear standard qPCR. Retrospective analysis by linear standard qPCR indicated that 40% of the patients in Part 1 received the 1.33 × 10^14^ vg/kg dose (30% of patients received 8.94 × 10^13^ vg/kg and 30% of patients received 6.29 × 10^13^ vg/kg). All patients treated with delandistrogene moxeparvovec in Part 2 received the 1.33 × 10^14^ vg/kg dose, as determined by linear standard qPCR.

### Sample collection

Biopsies from the medial gastrocnemius, or alternatively allowed muscle groups, were collected using open or VACORA^®^ core biopsies in accordance with processing protocols and transferred frozen to the sponsor laboratory.

### Western blot analysis of SRP-9001 dystrophin protein

Western blots were performed under Good Clinical Laboratory Practice standards, according to validated methodology adapted from (Charleston et al., 2018). Briefly, total protein was assayed in homogenized biopsied samples. Twenty micrograms of total protein per sample were loaded alongside negative controls and a 5-point standard curve (recombinant SRP-9001 protein [Curia, United States] ranging from 21.85 to 349.58 fmol/mg protein) in SDS-PAGE (Invitrogen, United States). Membranes with transferred proteins were probed with DYS3 primary antibody (1:20, Leica Biosystems, Germany), then anti-mouse IgG-conjugated horseradish peroxidase (1:1,000, GE Healthcare, United States). A chemiluminescence imaging system (Alliance Q9 Advanced Imager, UVITEC, United Kingdom) was used to visualize bound enzyme activity and the bands were analyzed using Image Quant TL Plus software (GE Healthcare, United States). For quantification of SRP-9001 dystrophin protein in each sample, data were normalized to each patient’s muscle content. Control samples were kindly provided by Dr. Steven A. Moore, Wellstone Center, University of Iowa, United States.

### IF analysis of SRP-9001 dystrophin protein

SRP-9001 dystrophin expression was analyzed by indirect IF staining using the following antibodies: anti-dystrophin (DYS3, Leica Biosystems, United Kingdom) at 1.4 μg/mL for 60 min and anti-laminin 2 alpha (laminin 2 alpha, Abcam, United Kingdom) at 5.5 μg/mL for 60 min. The appropriate secondary antibody cocktail (Thermo Scientific, United States) was used at 5 μg/mL for 30 min to detect dystrophin (Alexa Fluor 594) and laminin 2 alpha merosin (Alexa Fluor 488). Slides were scanned using a 3DHISTECH Pannoramic MIDI fluorescent scanner (PerkinElmer, United States) for a fixed exposure time. Scanned images were marked for regions of inclusion and exclusion (tissue folds, staining artifacts, etc.) using Flagship Biosciences (NC, United States) proprietary software. Individual muscle fibers were identified using a machine learning algorithm, based on laminin 2 alpha IF staining of the muscle membrane, and quantification of the dystrophin localized on each muscle fiber membrane was carried out.

### Vg quantification

SRP-9001 vector genome copies were quantified in muscle biopsies at baseline and 12 weeks post-treatment by digital droplet PCR (ddPCR), using a primer probe set targeting the MHCK7 promoter. Results were reported as vector genome copies per nucleus.

### Study outcomes and assessments

The primary endpoints were CFBL to Week 12 (Part 1) in SRP-9001 dystrophin protein, as measured by Western blot, and to Week 48 (Part 1) in NSAA total score. Secondary endpoints were CFBL to Week 48 (Part 1) in timed function tests (10-m Walk/Run [10MWR], 100MWR, 4-stair Climb, and supine to stand [Time to Rise]) and to Week 12 (Part 1) in SRP-9001 dystrophin protein expression, as measured by IF fiber intensity and IF PDPF. Safety outcomes were assessed by adverse events (AEs) and changes in laboratory parameters. Exploratory endpoints included CFBL to Week 12 (Part 2) in SRP-9001 dystrophin protein expression, as measured by Western blot, IF fiber intensity, and IF PDPF, and assessment of vector genome copy number by ddPCR to confirm successful SRP-9001 expression at Week 12 of Parts 1 and 2.

### Statistical analysis

Analyses were performed after the completion of Part 1 (primary) and Part 2 (exploratory) of the study. For the primary biologic endpoint (CFBL to Week 12 [Part 1] in SRP-9001 dystrophin expression, as measured by Western blot), a re-randomization test was performed using a two-sample Welch t-test as the test statistic. The t-test statistic was estimated using 10,000 re-randomization datasets, based on the observed dataset. For the primary functional endpoint (CFBL to Week 48 [Part 1] in NSAA score), a restricted maximum likelihood (REML)-based mixed model for repeated measures was used to compare treatment groups. In this model, the response variable consisted of the CFBL in NSAA score at each post-baseline visit in Part 1. The model included the covariates of treatment group (categorical), visit (categorical), treatment-group-by-visit interaction, age group (categorical), baseline value, and baseline-value-by-visit interaction. The treatment difference in CFBL was tested at a 2-sided statistical significance level of 0.05. The secondary endpoints were also analyzed, using the mixed model for repeated measures. For other endpoints, data analyses are primarily descriptive in nature.

As specified in the statistical analysis plan for the EC cohort, prior to unblinding of data, an analysis was conducted to contextualize Part 2 data with a propensity-score-weighted EC cohort that included patients from the Finding the Optimum Regimen for DMD (FOR-DMD) study (Clinicaltrials.gov, 2016), the Cooperative International Neuromuscular Research Group DMD Natural History Study (CINRG/DNHS) (Clinicaltrials.gov, 2012; Spurney et al., 2014), and the Eli Lilly tadalafil study (NCT01865084) (Clinicaltrials.gov, 2013; Victor et al., 2017). Propensity-score weighting was based on key prognostic factors in DMD, including age and baseline scores for NSAA and key timed function tests (Time to Rise and 10MWR) (Goemans et al., 2016; Goemans et al., 2020a; Goemans et al., 2020b; Naarding et al., 2020; Muntoni et al., 2022a; Muntoni et al., 2022b). LSM changes [standard error (SE)] with *p*-values are presented where modeling and comparison were conducted. Otherwise, descriptive means (standard deviation) are presented.

### EC cohort selection

To ensure that the clinical characteristics of the EC cohort were consistent with the baseline characteristics of the patients in this study, indicated external studies and/or registries were selected based on the following inclusion criteria at baseline: 4–8 years of age, inclusive; NSAA score ≥13 and ≤30; Time to Rise ≤10.4 s; 10MWR ≤9.1 s; and on a stable dose or dose-equivalent of oral corticosteroids for ≥12 weeks (patients on 10-day-on/10-day-off regime were excluded).

## Results

### Patient baseline characteristics

**FIGURE 1:**
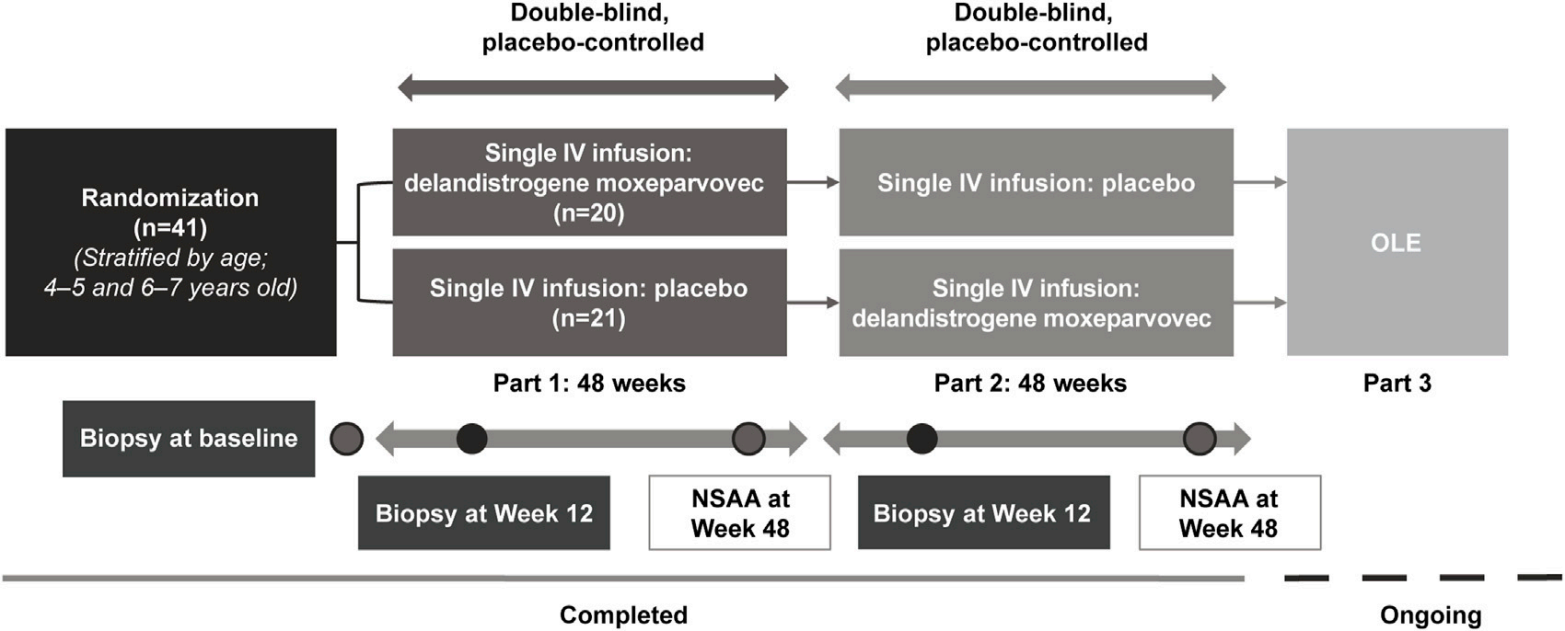
Study design. All patients in Part 1 received 2.0 × 10^14^ vg/kg, as determined by the supercoiled standard qPCR method specified in the protocol at the time. The 2.0 × 10^14^ vg/kg dose was estimated by supercoiled qPCR and is equivalent to 1.33 × 10^14^ vg/kg using the linear qPCR method. Retrospective analysis of the treatment lots by linear qPCR, the method utilized in Part 2 and subsequent studies of delandistrogene moxeparvovec, found variability in the doses administered in Part 1, such that: 30% (6/20) of patients received 8.94 × 10^13^ vg/kg, 30% (6/20) of patients received 6.29 × 10^13^ vg/kg, 40% (8/20) of patients received the correct linear equivalent of 2.0 × 10^14^ vg/kg delandistrogene moxeparvovec, 1.33 × 10^14^ vg/kg, and 100% (21/21) of patients treated in Part 2 received 1.33 × 10^14^ vg/kg. IV, intravenous; NSAA, North Star Ambulatory Assessment; OLE, open-label extension; qPCR, quantitative polymerase chain reaction.

**FIGURE 2:**
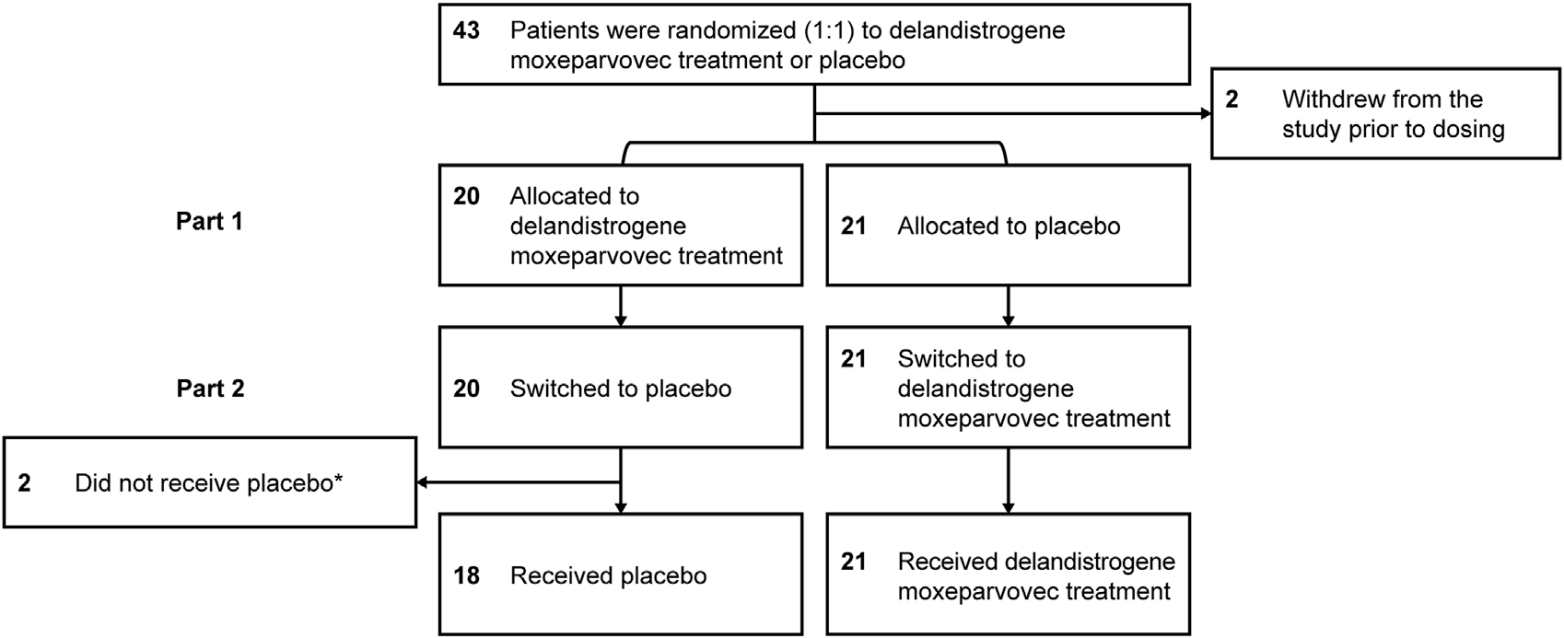
Patient disposition. *Two patients did not receive placebo in Part 2: one patient had to be tapered off of steroids due to a steroid-related AE and one patient required elective surgery for a femur fracture that required significant recovery time; both patients continue to be followed for efficacy and safety. AE, adverse event.

Forty-one eligible patients with DMD, ≥4 to <8 years of age, were enrolled and randomized to either the placebo or delandistrogene moxeparvovec treatment group (Figure 1). Twenty patients received a single IV administration of delandistrogene moxeparvovec, and 21 received placebo in Part 1. Patient disposition data are shown in Figure 2. All 41 participants completed Part 1 of the study. A total of 39 participants were infused with either delandistrogene moxeparvovec or placebo in Part 2. All 21 participants who had received placebo in Part 1 received delandistrogene moxeparvovec in Part 2. Of the 20 participants treated with delandistrogene moxeparvovec in Part 1, 18 received placebo in Part 2, and two patients did not: one patient had to be tapered off steroids due to a steroid-related AE and another patient required elective surgery for a femoral fracture, unrelated to treatment, which involved significant recovery time. Both patients continue to be followed for efficacy and safety in the open-label extension phase of the study.

The mean ages of patients treated with delandistrogene moxeparvovec or placebo in Part 1 were 6.3 and 6.2 years, respectively (Table 1). A majority of patients (61%) were ≥6 years of age at baseline. All participants were receiving steroids prior to enrollment, with 20/41 (48.8%) participants on a daily steroid regime, mostly oral prednisolone (Supplementary Table S1). Steroid types and frequency of use were balanced between the treatment groups at baseline in Part 1.

**TABLE 1:** Baseline characteristics of the overall population^a^.

Characteristic	Patients treated with delandistrogene moxeparvovec in Part 1^b^ (n = 20)	Patients treated with placebo in Part 1^c^ (n = 21)
Age (years)^d^		
Mean (SD)	6.29 (1.19)	6.24 (1.13)
Range	4.47–7.85	4.34–7.98
Median	6.52	6.03
Years since corticosteroid treatment started		
Mean (SD)	0.99 (1.07)	1.26 (1.22)
Range	0.22, 3.80	0.23, 5.07
Median	0.56	0.63
Corticosteroid type, deflazacort		
n (%)	7 (35.0)	7 (33.3)
Dosing weight (kg)		
Mean (SD)	23.28 (4.37)	21.60 (3.49)
Range	18.0–34.5	15.0–30.0
Median	22.50	21.50
NSAA total score^e^		
Mean (SD)	19.8 (3.3)	22.6 (3.3)
Median	20.0	22.0
Time to Rise (seconds)		
Mean (SD)	5.10 (2.17)	3.56 (0.65)
Median	4.30	3.40
4-stair Climb (seconds)		
Mean (SD)	3.69 (1.46)	3.10 (0.98)
Median	3.30	3.00
100MWR (seconds)		
Mean (SD)	61.04 (12.71)	53.86 (8.30)
Median	57.10	55.60
10MWR (seconds)		
Mean (SD)	5.35 (1.14)	4.83 (0.72)
Median	5.00	4.70

^a^ Intent-to-treat population.

^b^ Patients who received delandistrogene moxeparvovec in Part 1 and placebo in Part 2.

^c^ Patients who received placebo in Part 1 and delandistrogene moxeparvovec in Part 2.

^d^ The majority of patients (61%) were ≥6 years of age at baseline, and age was the only stratification factor for randomization.

^e^ The 4- to 5-year-old subgroup was well matched at baseline; however, in the 6- to 7-year-old subgroup, NSAA scores were not well matched at baseline.

Mutation type in treated and placebo cohorts: whole-exon deletion, 15/20 and 16/21; whole-exon duplication, 1/20 and 2/21; premature stop codon, 1/20 and 2/21; small insertion/deletion, 0/20 and 1/21; other, 3/20 and 1/21, respectively.

10MWR, 10-m Walk/Run; 100MWR, 100-m Walk/Run; NSAA, North Star Ambulatory Assessment; SD, standard deviation.

Analysis of baseline motor function indicated a significant mismatch between the treatment and placebo groups, with the mean baseline NSAA score in the delandistrogene moxeparvovec group being lower than the placebo group (19.8 vs 22.6, respectively; Table 1), thus indicating that the treated group had more advanced DMD relative to the placebo group. Timed function tests at baseline demonstrated a similar disparity, with the treated group performing worse (e.g., the Time to Rise at baseline was 5.1 s for the delandistrogene moxeparvovec group vs 3.6 s for the placebo group).

Subgroup analysis by age indicated that the mean NSAA scores in the treatment and placebo arms of the 4- to 5-year-olds were well matched at baseline, with scores of 20.1 and 20.4 points, respectively (Table 2A). In contrast, analysis of the 6- to 7-year-olds showed a significant difference in baseline mean NSAA scores between the treatment (19.6 points) and placebo (24.0 points) groups (*p* = 0.0046; Table 2B). Similarly, disparities were observed in the older subgroup in baseline timed function tests (e.g., the mean Time to Rise at baseline was 5.9 s for the treated group vs 3.4 s for the placebo group).

**TABLE 2:** Baseline NSAA and timed function test scores by pre-specified age subgroup A) Analysis of the 4- to 5-year-old subgroup.

Motor function assessment	Age 4–5 years		
	Delandistrogene moxeparvovec (n = 8)	Placebo (n = 8)	*p*-value (vs placebo)
NSAA total score			
Mean (SD)	20.10 (1.9)	20.40 (2.7)	0.8318
Median	20.50	20.50	
100MWR (seconds)			
Mean (SD)	58.76 (7.1)	59.79 (8.2)	0.7925
Median	57.90	59.70	
4-stair Climb (seconds)			
Mean (SD)	3.46 (0.9)	3.48 (1.3)	0.9822
Median	3.50	3.00	
Time to Rise (seconds)			
Mean (SD)	3.89 (0.7)	3.76 (0.8)	0.7421
Median	3.70	3.75	
10MWR (seconds)			
Mean (SD)	5.01 (0.6)	5.24 (1.0)	0.5832
Median	5.05	5.10	
B) Analysis of the 6- to 7-year-old subgroup			

Motor function assessment	Age 6–7 years		
	Delandistrogene moxeparvovec (n = 12)	Placebo (n = 13)	*p*-value (vs placebo)
NSAA total score			
Mean (SD)	19.60 (4.1)	24.00 (2.9)	0.0046
Median	20.00	24.00	
100MWR (seconds)			
Mean (SD)	62.56 (15.5)	50.21 (6.2)	0.0219
Median	57.10	50.40	
4-stair Climb (seconds)			
Mean (SD)	3.83 (1.8)	2.86 (0.71)	0.0958
Median	3.20	3.00	
Time to Rise (seconds)			
Mean (SD)	5.91 (2.5)	3.44 (0.6)	0.0053
Median	5.05	3.40	
10MWR (seconds)			
Mean (SD)	5.58 (1.4)	4.58 (0.4)	0.0313
Median	5.00	4.60	

10MWR, 10-m Walk/Run; 100MWR, 100-m Walk/Run; NSAA, North Star Ambulatory Assessment; SD, standard deviation.

### Functional outcomes

#### Part 1 results: Week 48

**FIGURE 3:**
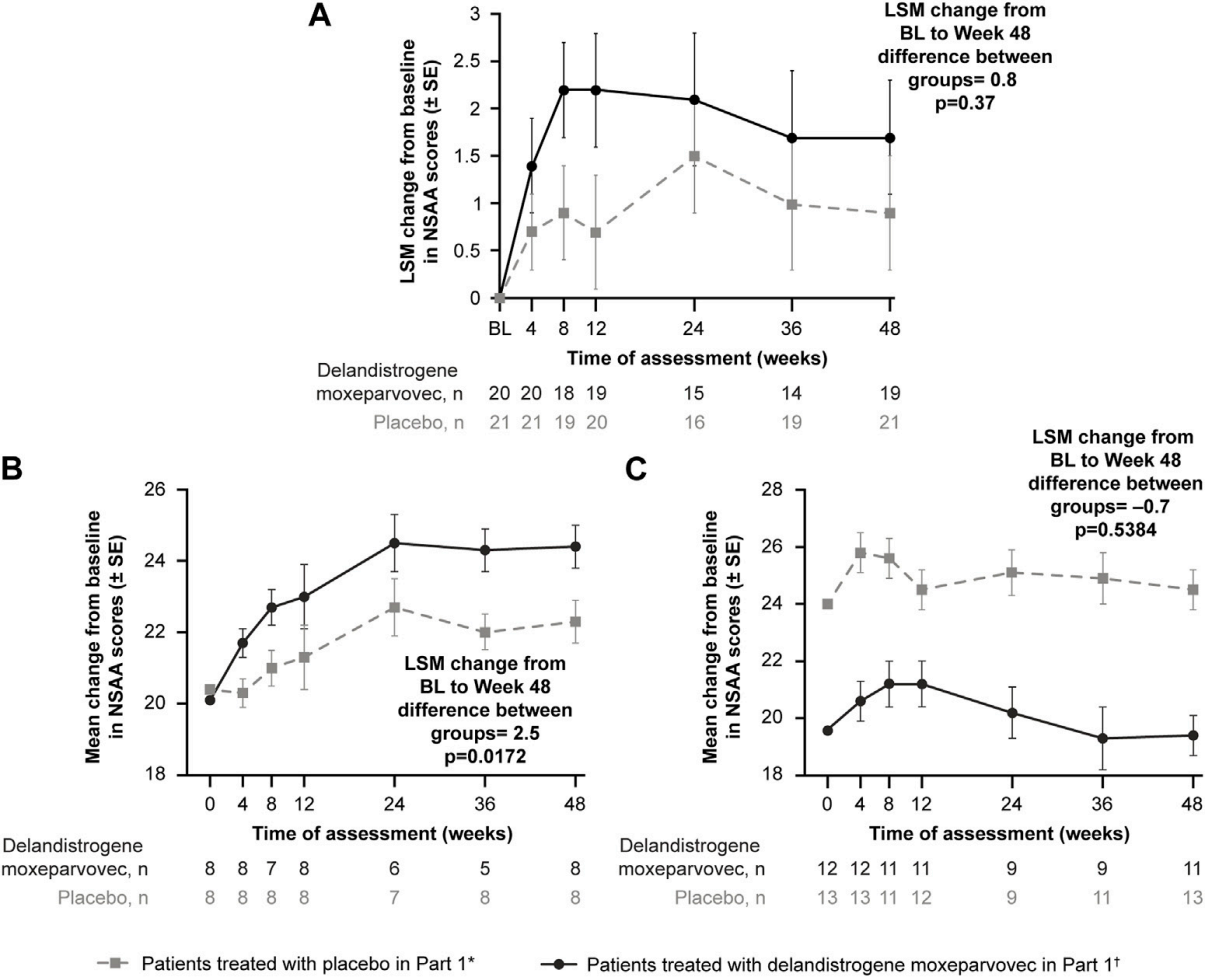
Primary functional endpoint: Change in NSAA score from baseline to Week 48 (Part 1) **(A)** depicts the CFBL to Week 48 in NSAA total score in the intent-to-treat population; **(B)** depicts the analysis of the 4- to 5-year-old subgroup; **(C)** depicts the analysis of the 6- to 7-year-old subgroup. *Patients who received placebo in Part 1 and delandistrogene moxeparvovec in Part 2. ^†^Patients who received delandistrogene moxeparvovec in Part 1 and placebo in Part 2. Two patients did not receive placebo in Part 2: one patient had to be tapered off of steroids due to a steroid-related AE and one patient required elective surgery for a femur fracture that required significant recovery time; both patients continue to be followed for efficacy and safety. AE, adverse event; BL, baseline; CFBL, change from baseline; LSM, least-squares mean; NSAA, North Star Ambulatory Assessment; SE, standard error.

At Week 48, the LSM change (SE) in NSAA score from baseline was +1.7 (0.6) points in the treatment group and +0.9 (0.6) points in the placebo group (Figure 3A); the between-group difference was 0.8 points (*p* = 0.37).

Analysis of the 4- to 5-year-old subgroup demonstrated a statistically significant LSM CFBL to Week 48 in NSAA scores of +2.5 points in the treatment versus placebo group (*p* = 0.0172; Figure 3B). Analysis of the 6- to 7-year-old subgroup, however, demonstrated an LSM CFBL to Week 48 in NSAA scores of −0.7 between the groups, which was not statistically significant (*p* = 0.5384; Figure 3C). Disparity in baseline motor function between groups likely confounded these results.

#### Part 2 results: Week 48 (Part 2) and Week 96

**FIGURE 4:**
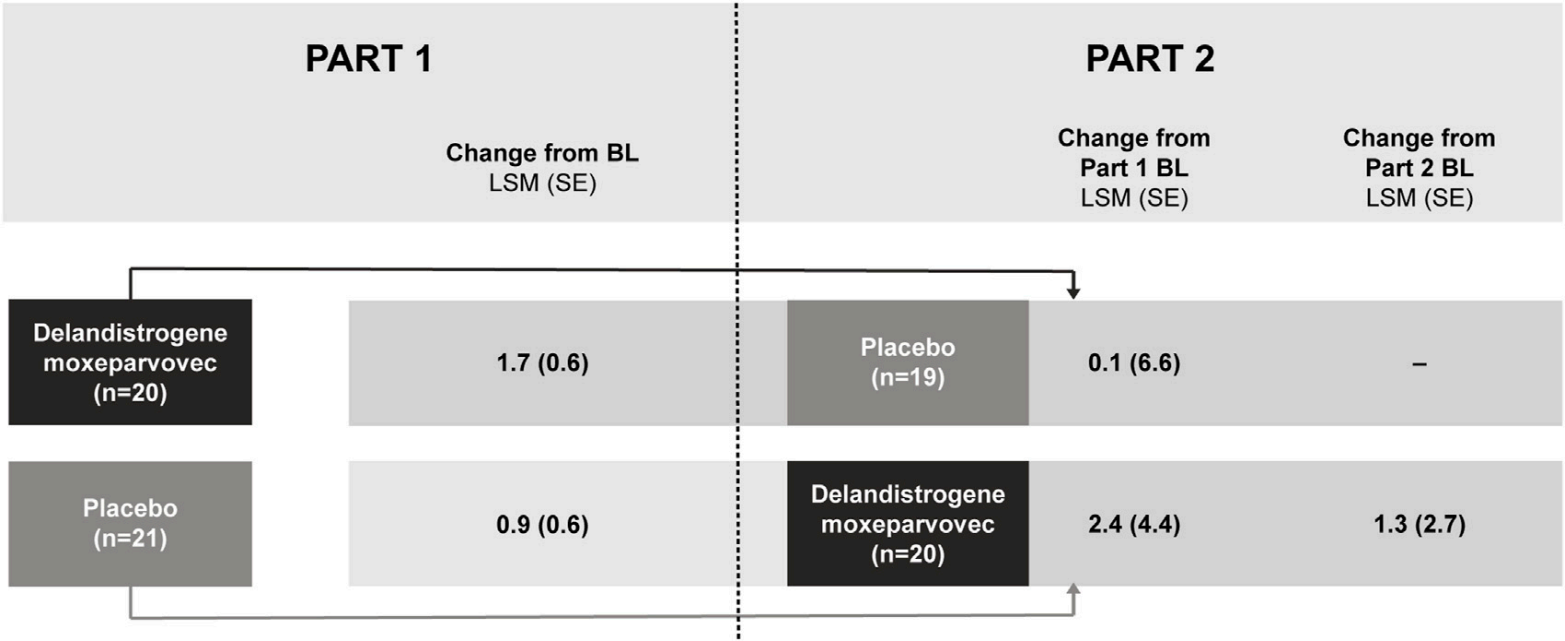
NSAA score change from baseline after treatment with delandistrogene moxeparvovec (Parts 1 and 2). Part 2 baseline values are given as this is when patients who were treated in Part 2 received delandistrogene moxeparvovec. BL, baseline; LSM, least-squares mean; NSAA, North Star Ambulatory Assessment; SE, standard error.

Patients who were randomized to delandistrogene moxeparvovec in Part 1 experienced a 96-week exposure to treatment by the end of Part 2. For these patients, the LSM (SE) CFBL to Week 96 in NSAA score was +0.1 (6.6) points (Figure 4). Of note, in this group there was an outlier with a significant 17-point drop in NSAA that may have skewed this result. For patients treated in Part 2, the LSM (SE) CFBL (Part 2) to Week 48 in NSAA score was +1.3 (2.7) points (Figure 4).

Results for the timed function tests can be found in Supplementary Table S2 and Supplementary Figure S1.

### Functional outcomes in patients treated with delandistrogene moxeparvovec compared with a propensity-score-weighted EC cohort

To contextualize the findings from Part 2, as all patients were exposed to delandistrogene moxeparvovec, results were compared with a pre-specified, propensity-score-weighted EC cohort. Baseline demographics of patients treated in Parts 1 and 2 versus the EC cohort are shown in Table 3.

**TABLE 3:** Baseline assessments of patients treated with delandistrogene moxeparvovec compared with matched, propensity-score-weighted EC cohorts for functional endpoints A) Part 1.

Baseline parameter	Delandistrogene moxeparvovec	EC
	(N = 19)	(N = 51)
Age (years)		
Mean (SD)	6.21 (1.17)	6.20 (0.45)
Median	6.49	6.10
NSAA total score		
Mean (SD)	19.9 (3.4)	19.7 (1.9)
Median	20.0	20.0
Time to Rise (seconds)		
Mean (SD)	5.17 (2.21)	5.22 (1.05)
Median	4.60	4.70
10MWR (seconds)		
Mean (SD)	5.39 (1.16)	5.39 (0.58)
Median	5.10	5.50
B) Part 2 (crossover)		

Baseline parameter	Delandistrogene moxeparvovec	EC
	(N = 20)	(N = 103)
Age (years)		
Mean (SD)	7.24 (1.12)	7.03 (0.42)
Median	7.07	6.97
NSAA total score		
Mean (SD)	23.8 (3.7)	23.5 (1.9)
Median	24.5	24.0
Time to Rise (seconds)		
Mean (SD)	4.02 (1.34)	3.92 (0.59)
Median	3.80	3.70
10MWR (seconds)		
Mean (SD)	4.84 (1.15)	4.83 (0.40)
Median	4.65	4.90

10MWR, 10-m Walk/Run; EC, external control; NSAA, North Star Ambulatory Assessment; SD, standard deviation.

### Patients treated in Part 1 versus EC cohort

**FIGURE 5:**
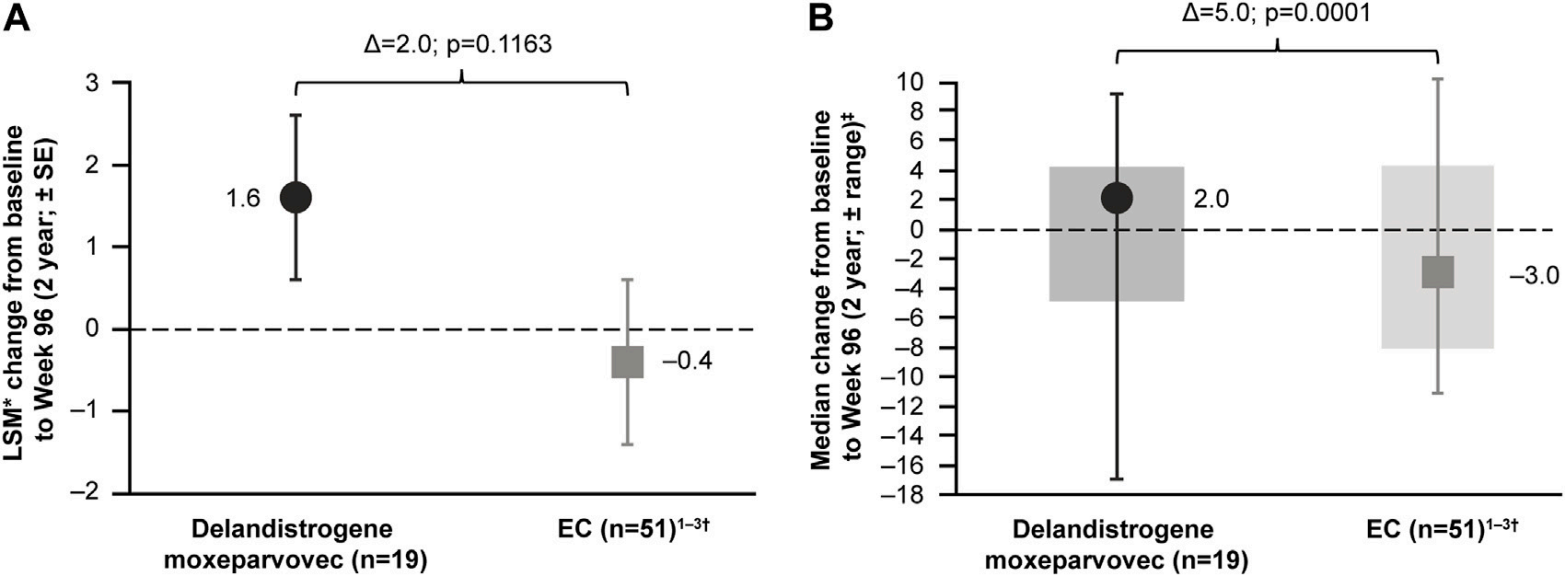
Patients treated with delandistrogene moxeparvovec in Part 1 versus EC cohort: NSAA analyses **(A)** shows the LSM* CFBL to Week 96 in NSAA total score for the delandistrogene moxeparvovec overall cohort treated in Part 1 versus EC cohort; **(B)** shows the median CFBL to Week 96 in NSAA total score for the delandistrogene moxeparvovec overall cohort treated in Part 1 versus EC; boxes represent interquartile range and bars represent the minimum and maximum range. *LSM from weighted linear regression. †For the 96-week (2-year) comparator group, EC data were only available for 51 participants. 1. Clinicaltrials.gov, 2012; Clinicaltrials.gov, 2013; Clinicaltrials.gov, 2016. CFBL, change from baseline; EC, external control; LSM, least-squares mean; NSAA, North Star Ambulatory Assessment; SE, standard error.

For patients treated in Part 1, the LSM CFBL to Week 96 in NSAA total score was +1.6 points compared with −0.4 points for the EC cohort, with a between-group difference of 2.0 points, which was not statistically significant (*p* = 0.1163; Figure 5A). It was thought that a patient with an outlying data point for NSAA (a 17-point decrease) may have skewed the mean estimate. This patient’s age at baseline, Time to Rise, and NSAA score were 7.6 years, 6.7 s, and 21 points, respectively. The data were therefore re-analyzed using the median NSAA scores and found to show an improvement over 2 years (median between-group difference of 5.0 points; *p* = 0.0001; Figure 5B).

### Patients treated in Part 2 versus EC cohort

**FIGURE 6:**
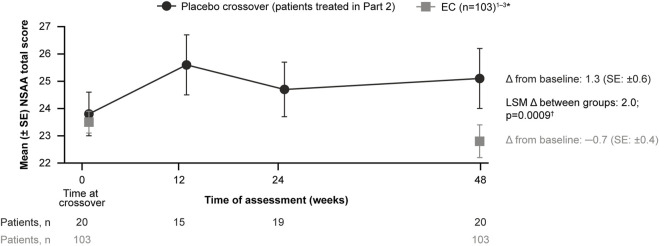
Patients treated with delandistrogene moxeparvovec in Part 2 versus EC cohort: NSAA analyses. The mean CFBL to Week 48 in NSAA total score was +1.3 points for patients treated in Part 2 versus −0.7 points for the EC cohort. *For the 48-week (1-year) comparator group, EC data were only available for 103 participants. †The *p*-value was calculated based on weighted ANCOVA adjusted for age and baseline NSAA (Clinicaltrials.gov, 2012; Clinicaltrials.gov, 2013; Clinicaltrials.gov, 2016). ANCOVA, analysis of covariance; CFBL, change from baseline; EC, external control; LSM, least-squares mean; NSAA, North Star Ambulatory Assessment; SE, standard error.

For patients treated with delandistrogene moxeparvovec in Part 2, the mean CFBL to Week 48 (Part 2) in NSAA total score was +1.3 points, compared with −0.7 points for the EC cohort (Figure 6). The LSM between-group difference was +2.0 points, which was statistically significant (*p* = 0.0009).

### Biologic outcomes (SRP-9001 dystrophin protein expression and SRP-9001 transgene delivery)

**FIGURE 7:**
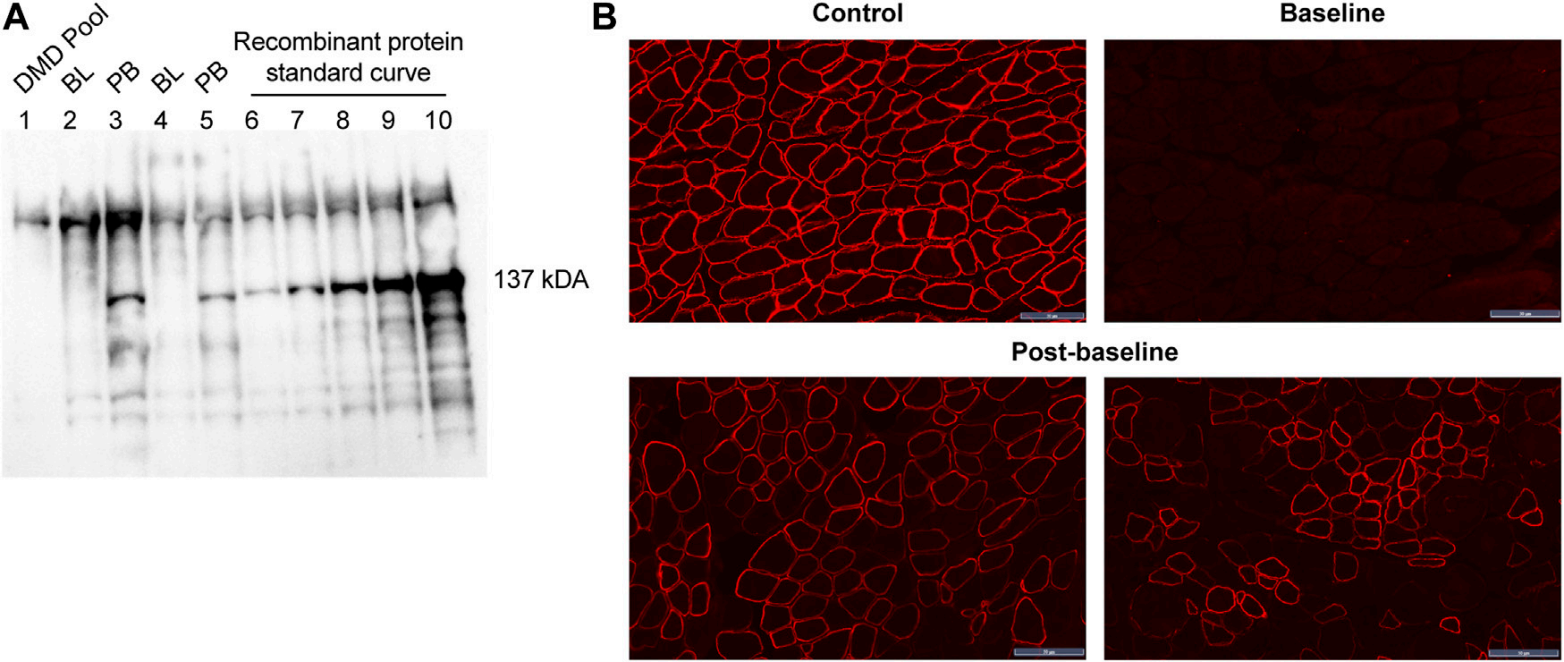
SRP-9001 dystrophin protein expression at 12 weeks post-infusion **(A)** shows a representative Western blot for SRP-9001 dystrophin. Lanes 1–5 indicate: DMD pool (negative control), BL, PB, BL, PB; Lanes 6–10: Recombinant micro-dystrophin protein standard curve (21.85, 43.70, 87.39, 174.79, 349.58 fmol/mg). The 137 kDa band denotes the presence of SRP-9001 dystrophin; **(B)** shows representative IF images of biopsied sections of gastrocnemius muscle stained with dystrophin antibody. Top panel: L to R: normal expression in control tissue, pre-treatment BL expression; Lower panel: select images of Week 12 post-baseline expression. Scale bar, 50 µm. BL, baseline; DMD, Duchenne muscular dystrophy; IF, immunofluorescence; PB, post-baseline.

All patients treated with delandistrogene moxeparvovec demonstrated robust expression of SRP-9001 dystrophin protein (Figure 7). Treatment with delandistrogene moxeparvovec resulted in correct localization of SRP-9001 dystrophin to the sarcolemma in a large proportion of muscle fiber, as demonstrated by IF PDPF and IF fiber intensity. In Part 1, quantification of SRP-9001 dystrophin showed a greater mean CFBL to Week 12 in treated patients compared with placebo by Western blot (23.82% vs 0.14%, *p* < 0.0001), IF fiber intensity (25.81% vs −0.48%, *p* = 0.0002), and IF PDPF (23.88% vs 5.09%, *p* = 0.0056) (Table 4A).

**TABLE 4:** Change from baseline in biologic outcomes for all patients.

A) Part 1							
			Delandistrogene moxeparvovec (n = 20)^a^		Placebo (n = 21)^b^	*p*-value	
			CFBL to Week 12		CFBL to Week 12		
Western blot adjusted for muscle content, % normal	Mean (SD)		23.82 (39.76)		0.14 (1.24)	<0.0001	
Vector genome copy number	Mean (SD)		1.56 (1.51)		0.00	<0.0001	
Fiber intensity, % control	Mean (SD)		25.81 (46.23)		−0.48 (6.29)	0.0002	
PDPF, %	Mean (SD)		23.88 (25.58)		5.09 (12.96)	0.0056	

B) Part 2 Week 12 (crossover)							
Placebo/delandistrogene moxeparvovec (n = 21)^†^							
CFBL to Week 12							
Western blot adjusted for muscle content, % normal	Mean (SD)		39.64 (31.79)				
	*p*-value		<0.0001				
Vector genome copy number	Mean (SD)		3.43 (2.02)				
	*p*-value		<0.0001				
Fiber intensity, % control	Mean (SD)		74.09 (47.69)				
	*p*-value		<0.0001				
PDPF, %	Mean (SD)		78.92 (22.47)				
	*p*-value		<0.0001				

C) Part 2 Week 12 (Part 1 Week 60) (crossover)							
Delandistrogene moxeparvovec/placebo (n = 18)^a^							
CFBL to Week 12 (Part 1 Week 60)							
Western blot adjusted for muscle content, % normal	Mean (SD)		19.10 (36.43)				
	*p*-value		0.0048				
Vector genome copy number	Mean (SD)		0.94 (1.25)				
	*p*-value		<0.0001				
Fiber intensity, % control	Mean (SD)		38.30 (62.83)				
	*p*-value		0.0014				
PDPF, %	Mean (SD)		57.12 (30.63)				
	*p*-value		<0.0001				

^a^ Patients who received delandistrogene moxeparvovec at all doses in Part 1 and placebo in Part 2. Two patients did not receive placebo in Part 2 and did not have the Part 2 (Week 60) biopsy: one patient had to be tapered off of steroids due to a steroid-related AE and one patient required elective surgery for a femur fracture that required significant recovery time; both patients continue to be followed for efficacy and safety.

^b^ Patients who received placebo in Part 1 and delandistrogene moxeparvovec at all doses in Part 2. AE, adverse event; CFBL, change from baseline; PDPF, percent dystrophin-positive fibers; SD, standard deviation.

Patients treated in Part 2 showed a mean increase in SRP-9001 dystrophin expression from baseline to Week 12 (Part 2) by Western blot (39.64%, *p* < 0.0001), IF fiber intensity (74.09%, *p* < 0.0001), and IF PDPF (78.92%, *p* < 0.0001) (Table 4B). Those treated in Part 1 continued to show a mean increase in SRP-9001 dystrophin expression from baseline to 60 weeks post-treatment by Western blot (19.10%, *p* = 0.0048), IF fiber intensity (38.30%, *p* = 0.0014), and IF PDPF (57.12%, *p* < 0.0001) (Table 4C).

Evaluation of vector genome copy number per nucleus confirmed successful delivery of the SRP-9001 transgene, as measured by ddPCR. In Part 1, treatment resulted in a mean increase of 1.56 vector genome copies from baseline to Week 12, compared with 0.00 copies for placebo (*p* < 0.0001) (Table 4A). In Part 2, treatment resulted in a mean increase of 3.43 vector genome copies from baseline to Week 12 (*p* < 0.0001), and for those treated in Part 1, a mean increase of 0.94 copies from baseline to Week 60 (*p* < 0.0001) was observed (Tables 4B,C).

### Safety

In Parts 1 and 2, the most common treatment-related AEs (TRAEs) were vomiting, decreased appetite, and nausea (Tables 5A,B). Most TRAEs were reported within the first 90 days post-treatment.

**TABLE 5:** Summary of AEs.

A) Part 1		
	Delandistrogene moxeparvovec^a^ (n = 20)	Placebo^b^ (n = 21)
Total number of AEs	308	230
Patients with at least one AE, n (%)	20 (100.0)	21 (100.0)
Total number of TEAEs	285	209
Patients with at least one TEAE, n (%)	20 (100.0)	21 (100.0)
Treatment-related TEAE, n (%)	17 (85.0)	9 (42.9)
Total number of SAEs	4	2
Patients with at least one SAE, n (%)	3 (15.0)	2 (9.5)
Treatment-related SAE, n (%)	3 (15.0)	1 (4.8)
Patients with an AE leading to study discontinuation, n	0	0
Deaths, n	0	0
Treatment-related SAEs, n (%)		
Rhabdomyolysis	2 (10.0)	1 (4.8)
Increased transaminases	1 (5.0)	0
Liver injury	1 (5.0)	0
Treatment-related TEAEs, n (%)^c^		
Vomiting	12 (60.0)	4 (19.0)
Decreased appetite	6 (30.0)	0
Nausea	6 (30.0)	2 (9.5)
Gamma-glutamyl transferase increased	5 (25.0)	0
Abdominal pain upper	3 (15.0)	1 (4.8)
Abdominal pain	3 (15.0)	0
Blood bilirubin increased	2 (10.0)	0
Pain in extremity	2 (10.0)	1 (4.8)
Rhabdomyolysis	2 (10.0)	1 (4.8)
Pyrexia	1 (5.0)	0

B) Part 2 (crossover)		
	Placebo^a^ (n = 20)	Delandistrogene moxeparvovec^b^ (n = 21)
Total number of AEs	157	278
Patients with at least one AE, n (%)	19 (95.0)	21 (100.0)
Total number of TEAEs	131	262
Patients with at least one TEAE, n (%)	19 (95.0)	21 (100.0)
Treatment-related TEAE, n (%)	4 (20.0)	20 (95.2)
Total number of SAEs	2	1
Patients with at least one SAE, n (%)	2 (10.0)	1 (4.8)
Treatment-related SAE, n (%)	0	0
Patients with an AE leading to study discontinuation, n	0	0
Deaths, n	0	0
Treatment-related TEAEs, n (%)^c^		
Vomiting	0	16 (76.2)
Decreased appetite	0	15 (71.4)
Nausea	1 (5.0)	10 (47.6)
Gamma-glutamyl transferase increased	0	6 (28.6)
Abdominal pain upper	1 (5.0)	8 (38.1)
Abdominal pain	0	1 (4.8)
Blood bilirubin increased	0	2 (9.5)
Pyrexia	0	4 (19.0)
Thrombocytopenia	0	5 (23.8)
Glutamate dehydrogenase increased	0	3 (14.3)
Fatigue	0	2 (9.5)
Headache	0	2 (9.5)
Ketonuria	1 (5.0)	1 (4.8)
Lethargy	0	2 (9.5)
Myalgia	0	2 (9.5)
White blood cell count decreased	0	2 (9.5)

^a^ Patients who received delandistrogene moxeparvovec in Part 1 and placebo in Part 2. Two patients did not receive placebo in Part 2: one patient had to be tapered off of steroids due to a steroid-related AE and one patient required elective surgery for a femur fracture that required significant recovery time; both patients continue to be followed for efficacy and safety. Data represent AEs experienced in second year post-treatment.

^b^ Patients who received placebo in Part 1 and delandistrogene moxeparvovec in Part 2.

^c^ Treatment-related TEAEs reported in at least two patients in Part 1 or Part 2.

AE, adverse event; SAE, serious AE; TEAE, treatment-emergent AE.

Five treatment-related serious AEs (TR-SAEs) were reported in Part 1 (Table 5A). There were three instances of rhabdomyolysis (two patients who received delandistrogene moxeparvovec and one patient who received placebo) that resolved. Increased transaminases were reported in one patient and liver injury was reported in another (both in patients who received delandistrogene moxeparvovec), which resolved. No TR-SAEs were reported during Part 2 of the study (Table 5B).

There were no deaths or study discontinuations due to an AE. Serious events related to complement activation were not seen and there were no serious abnormalities observed in hematologic and chemistry panels. No new safety signals emerged for patients treated in Part 1, 2 years post-infusion.

## Discussion

Our findings support that delandistrogene moxeparvovec has a favorable benefit–risk profile and confirm robust expression of SRP-9001 dystrophin protein following treatment. These results build on our understanding of delandistrogene moxeparvovec from the Phase 1 trial, which demonstrated initial improvement and durable, sustained stabilization of motor function over 4 years post-treatment (mean age at Year 4 of 9.2 years); manuscript under review. Furthermore, we have observed a consistent and manageable safety profile in this and other open-label clinical studies of delandistrogene moxeparvovec (SRP-9001-101 [NCT03375164] and ENDEAVOR [SRP-9001-103; NCT04626674]; manuscripts under review) to date, with most TRAEs occurring within the first 90 days post-treatment.

The effect of delandistrogene moxeparvovec on motor function was assessed by the NSAA over 48 weeks (Part 1) as a pre-specified co-primary endpoint. Treatment with delandistrogene moxeparvovec resulted in improved NSAA total scores in Part 1; however, the difference between the treatment and placebo groups was not statistically significant, likely because these cohorts were stratified solely on age at randomization, which did not match important prognostic differences in motor function between the two groups. Indeed, there was a significant imbalance in baseline motor function scores between the treatment and placebo groups in the 6- to 7-year-old subgroup, wherein patients randomized to placebo in Part 1 had far less progressed disease, relative to those randomized to receive delandistrogene moxeparvovec. For heterogeneous diseases, like DMD, randomized controlled trials should utilize cohorts that are well matched across important baseline and prognostic variables in order to account for sources of heterogeneity (Goemans et al., 2016; Goemans et al., 2020a; Goemans et al., 2020b; Naarding et al., 2020; Muntoni et al., 2022b).

Subgroup analysis of patients in Part 1 showed that 4- to 5-year-olds were well matched for motor function at baseline in the treatment and placebo groups. In this subgroup, a statistically significant and clinically meaningful difference of +2.5 points was observed in the LSM CFBL to Week 48 in NSAA scores. In contrast, in the 6- to 7-year-old subgroup, baseline motor function differed significantly in the treatment and placebo groups, and thus, the between-group difference in the CFBL to Week 48 in NSAA scores was not significant. Typically, NSAA scores peak in patients with DMD at a mean age of 6–7 years, after which they plateau and subsequently decline at a rate of roughly 3 points per year (Muntoni et al., 2019).

Motor function stabilized for up to 2 years following administration of delandistrogene moxeparvovec in this population of ambulatory patients (mean age at start of treatment: 6.3 years for patients treated in Part 1 and 7.2 years for patients treated in Part 2). Functional decline in individuals with DMD is expected after ∼6.3 years of age, based on natural history studies, including patients receiving standard-of-care corticosteroid therapy (Muntoni et al., 2019). Consistent with this, when comparing patients treated in Part 2 with a well-matched, propensity-score-weighted EC cohort, a statistically significant difference in the relative 48-week LSM change in NSAA score was observed. While patients treated in Part 1 demonstrated a higher mean NSAA score compared with the EC cohort at 96 weeks post-treatment, this difference was not statistically significant. To assess whether an outlier, with a significant 17-point drop, may have skewed this result, these data were reanalyzed using the median NSAA total score. This reanalysis subsequently showed a statistically significant difference between treated patients and the EC cohort.

SRP-9001 dystrophin expression at 12 weeks (Part 1) post-infusion, as measured by Western blot of biopsied muscle tissue, was a co-primary efficacy endpoint in this study. Robust expression of SRP-9001 dystrophin protein was shown in the biopsies of all patients at 12 weeks post-treatment with delandistrogene moxeparvovec, as evidenced by sarcolemmal localization of dystrophin protein, quantitative assessment of western blots, and vector genome copies, confirming successful delivery of the SRP-9001 transgene to target cells. These biologic results are consistent with previous studies (Mendell et al., 2020). Variability in the doses of delandistrogene moxeparvovec administered to patients treated in Part 1 versus Part 2 may have contributed to the differences in observed expression levels at Week 12 (23.82% normal for patients treated in Part 1 vs 39.64% normal for patients treated in Part 2).

Notably, patients treated in Part 2 of the study, who were an average of 7.2 years of age, demonstrated improvement or stabilization of motor function, suggesting that delandistrogene moxeparvovec may be beneficial to a range of patients, even those in predicted stages of functional decline.

The safety profile of delandistrogene moxeparvovec was consistent in both parts of this study. The most common TRAE was vomiting, as has been seen in the Phase 1 study. Most TRAEs occurred early and resolved within the first 90 days post-infusion. Serious events related to complement activation were not seen and no new safety signals were identified.

A significant limitation of this study is that at randomization patients were stratified by age alone, despite the known heterogeneity of the disease. Specifically, the variability in motor function at baseline was not considered, which likely confounded the primary functional analysis at Week 48. In addition, not all participants received 1.33 × 10^14^ vg/kg dosing as determined by linear qPCR. Furthermore, although a well-matched, propensity-score-weighted EC was used to contextualize the results, unmeasured confounding variables could still be present following propensity-score weighting, such as the possible difference in the duration and intensity of steroid use between patients in this study and the EC cohort.

## Conclusion

Overall, the safety profile of delandistrogene moxeparvovec in this Phase 2 study was consistent with the previous Phase 1 study, suggesting that it has a favorable benefit–risk profile. Stabilization in motor function following a single administration of delandistrogene moxeparvovec was sustained over 2 years in this population of ambulatory patients aged ≥4 to <8 years. Importantly, this functional stabilization was observed at a time when functional decline is expected, based on natural history. Robust SRP-9001 dystrophin protein expression was observed up to 60 weeks post-treatment. Further studies are ongoing to assess the safety and efficacy of delandistrogene moxeparvovec in broader populations of patients with DMD using the intended commercial process material, which include ENDEAVOR (NCT04626674) (Clinicaltrials.gov, 2022a), a Phase 1b study, and EMBARK (NCT05096221) (Clinicaltrials.gov, 2022b), a larger Phase 3 study. A plain language summary of the data reported in this manuscript is available in the Supplementary Materials.

## Data Availability

The datasets presented in this article are not readily available. Qualified researchers may request access to the data that support the findings of this study from Sarepta Therapeutics, Inc., Cambridge, MA, United States, by contacting medinfo@sarepta.com. Requests to access the datasets should be directed to Sarepta Therapeutics, medinfo@sarepta.com.
