# Supplementary material for: Expression of SRP-9001 dystrophin and stabilization of motor function up to 2 years post-treatment with delandistrogene moxeparvovec gene therapy in individuals with Duchenne muscular dystrophy
Source: Front Cell Dev Biol. 2023 Jul 11;11:1167762. doi: 10.3389/fcell.2023.1167762 (PMC10366687; doi:10.3389/fcell.2023.1167762)
Supplement: Supplementary file 1 [file DataSheet2.docx]

**Expression of SRP-9001 dystrophin and stabilization of motor function up to 2 years post-treatment with** **delandistrogene moxeparvovec gene therapy in individuals with Duchenne muscular dystrophy**

**Plain language abstract (Words: 549)**

Duchenne muscular dystrophy (DMD) is a rare, genetic disease that mostly affects boys. DMD is caused by changes (known as mutations) in a gene—the *DMD* gene—that contains the instructions for making a muscle protein, called dystrophin. These mutations result in the absence of functional dystrophin protein. Dystrophin is important for protecting muscles from damage during normal movement, including during normal lung and heart function. DMD is a progressive disease, meaning muscles throughout the body weaken over time. There is no cure for DMD, but treatments in development offer new hope for patients living with the disease.

Delandistrogene moxeparvovec is a gene therapy designed to replace the missing dystrophin protein by delivering an engineered version of the *DMD* gene—*SRP-9001 dystrophin*—to muscle in people with DMD. To evaluate the safety and effectiveness of delandistrogene moxeparvovec, a clinical study was undertaken in boys with DMD, aged 4–7 years, who were still able to walk independently. The study was made up of two parts. In the first part of the study, which was 48 weeks long, 20 boys were given delandistrogene moxeparvovec while 21 boys received non-treatment (called placebo). After completing Part 1, for the second part of the study (also 48 weeks long), participants who received placebo in Part 1 were given delandistrogene moxeparvovec and those who received delandistrogene moxeparvovec in Part 1 were given placebo. The study was conducted in such a way that participants and their care team were both unaware of which treatments were given in each part. This process is called double-blinding.

In this study, researchers found that a single treatment with delandistrogene moxeparvovec led to an increase in SRP-9001 dystrophin protein in muscle, suggesting that the engineered dystrophin gene was able to get into these cells, allowing them to make the engineered dystrophin protein. Vomiting, decreased appetite, and nausea were the most common treatment-related side effects. Most occurred within the first 90 days and all resolved. Although delandistrogene moxeparvovec gene therapy was not associated with many serious side effects, there were two instances of rhabdomyolysis (muscle injury), one instance of increased transaminases (a liver enzyme), and one instance of liver injury, which all resolved. The effect of treatment on physical function was assessed using a scale called the North Star Ambulatory Assessment (NSAA) which includes 17 items (including walking/running 10 meters, getting up from the floor, and other standing and walking tasks), and other tests like the 100-meter timed test and climbing stairs. The study did not detect a significant difference in NSAA between the boys who were treated with gene therapy and those who received placebo in the overall population at 48 weeks post-treatment. The researchers found that there was a mismatch in disease severity between the two groups, despite random assignment of participants to treatment or placebo. As a result, boys who received placebo in Part 1, entered the study with higher NSAA scores on average, which affected meaningful comparison of results between boys who received the gene therapy and those who received placebo. Overall, researchers concluded that, on average, physical functioning remained generally stable for up to 2 years following a single administration of the gene therapy in this population of patients. This finding is encouraging because people with DMD typically begin to lose muscle function at this age.
